# Genetic landscape of stage II melanoma identifies *CBL* as a new driver gene and prognostic biomarker

**DOI:** 10.1038/s41416-026-03394-1

**Published:** 2026-04-09

**Authors:** Elena Sophia Lindner, Jakob Admard, German Demidov, Sorin Armeanu-Ebinger, Tobias Sinnberg, Heike Niessner, Teresa Amaral, Claus Garbe, Andrea Forschner, Olga Kelemen, Franz Joachim Hilke, Irina Bonzheim, Martin Röcken, Olaf Rieß, Stephan Ossowski, Christopher Schroeder

**Affiliations:** 1 Institute of Medical Genetics and Applied Genomics, University of Tübingen, Tübingen, Germany; 2 Center for Dermatooncology, Department of Dermatology, University Hospital Tübingen, Tübingen, Germany; 3 Charité Universitätsmedizin Berlin, Department of Dermatology, Venereology and Allergology, Berlin, Germany; 4 Institute of Pathology and Neuropathology, Liebermeisterstr. 8, University Hospital Tübingen, Tübingen, Germany; 5 Institute for Bioinformatics and Medical Informatics (IBMI), University of Tübingen, Tübingen, Germany

**Keywords:** Next-generation sequencing, Prognostic markers

## Abstract

**Background:**

While adjuvant therapies are currently recommended for patients with stage IIB-IV melanoma, biomarkers are missing to improve risk stratification to select the most efficient treatment and patients to be included in clinical trials. We genetically characterized a large cohort of patients with stage-II-melanoma to identify predictive and prognostic biomarkers.

**Methods:**

Clinical data of 193 stage-II-melanoma patients from the German Central Malignant Melanoma Registry were identified. All patients were therapy-naïve at the time of primary resection and received no adjuvant treatment until recurrence. Tumour-normal pairs were sequenced with a comprehensive cancer panel.

**Results:**

30.1% of the tumours were classified as *BRAF*-mutated, 28.0% as *RAS-*, 18.1% as *NF1-*, and 23.8% as Triple-WT. In-silico prediction identified a potential new candidate driver, *CBL*, in 10.4% of the patients. GISTIC nominated deletions of region 11q23.1-3 containing *CBL* as a potential driver alteration. Enrichment of mutations in *CBL* was replicated in published cohorts. Patients with *RAS*-mutated tumours and *CBL* deletions showed worse OS (*p* = 0.004) and RFS (*p* = 0.044). Point mutations in *CBL* were highly enriched in patients with *NF1*-mutated tumours, showing a trend (*p* = 0.18) towards worse OS.

**Conclusions:**

Our findings suggest *CBL* as a novel driver gene in melanoma, which is mutated in a relevant fraction of patients. Deletions of 11q23.1-3 including *CBL* were identified as a prognostic marker indicating a higher risk of recurrence and shorter survival. Furthermore, *CBL* could support patient stratification to identify high-risk patients who may benefit from adjuvant therapies or intensified monitoring strategies.

## Background

Analyses of the genetic landscape of cutaneous melanoma led to the identification of four molecular subtypes, which are mainly based on driver mutations in the MAPK signalling pathway: *BRAF*-mutated, *RAS-*, *NF1-* and Triple-WT melanoma [1]. Additional driver genes, like *CDKN2A*, *PTEN*, *MAP2K1*, *PPP6C* and *ARID2*, were added by functional or statistical analyses of genes recurrently mutated or affected by somatic copy number alterations [2–7]. Other characteristics of cutaneous melanoma genomes are a high overall tumour mutational burden associated with UV signature pointing to an important role of sun exposure in tumour development [1, 8].

The incidence of melanoma increased over the last two decades, with a simultaneous shift to earlier stages at diagnosis [9, 10]. While 10 years survival rates are good for AJCC stage IIA melanoma, stages IIB and IIC have significantly lower melanoma-specific survival rates [11]. Adjuvant anti-PD-1 therapy showed a benefit in relapse-free and distant metastases free survival in patients with stage IIB/C melanoma, but overall survival data is still immature. Both nivolumab and pembrolizumab are approved by FDA and EMA in this setting, but the reimbursement scenario is quite heterogenous [12]. Genetic biomarkers that could help to identify patients who benefit from this treatment are still missing. Interestingly, while classification of melanoma is based on the aforementioned driver genes, their prognostic significance is still discussed controversially [1, 13–16]. This adds to the financial burden on one side but also exposes patients to potential irreversible immune-related toxicity [17].

Here, we describe the genetic landscape of a large cohort of stage II melanoma patients, confirm known drivers, and identify a new prognostic genetic marker for RFS and OS in patients with stage II melanoma and *RAS* mutations. Furthermore, we discuss the potential of this marker to aid in the stratification of high-risk patients who might benefit most from adjuvant therapies, providing a step towards personalized treatment approaches in stage II melanoma.

## Methods

### Clinical specimen

Patients diagnosed with stage II melanoma were identified using data from the Central Malignant Melanoma Registry (CMMR). Since a sentinel lymph node biopsy has been a standard procedure in our clinic since 2000, we focused on the period between 2000 and 2018 to ensure correct AJCC staging [18]. Next, we identified the patients from whom FFPE samples of the primary tumour were available. All patients included in this study were therapy-naïve at the time of sample collection, receiving no adjuvant treatment until recurrence. All patients gave their informed consent for collecting their data in the CMMR and the study was approved by the local ethics committee (project numbers: 062/2019BO2, 396/2019BO2). Patients were treated with excision of the primary tumour according to German therapy guidelines.

The tumour was identified in a H&E-stained slide by a certified dermatopathologist and the diagnosis was confirmed. To isolate tumour and normal DNA for next-generation sequencing, microdissection of tissue sections was performed. DNA from tumour-normal pairs was isolated from 8 µm tumour and normal tissue paraffin sections using the Maxwell® RSC DNA FFPE Kit and the Maxwell® RSC Instrument (Promega, Madison, WI, USA) according to the manufacturer’s instructions.

### Library construction, hybrid capture, next-generation sequencing

A total of 200 ng of extracted genomic DNA (gDNA) was used for library construction. Genomic DNA was fragmented to 150-200 base pairs (bp) using Covaris Ultrasonication (LE220 Focused-ultrasonicator, Covaris, Woburn, MA, USA). The SureSelect XT Low Input Target Enrichment System (Agilent Technologies, Santa Clara, CA, USA) was used to perform hybrid capture with custom-designed bait sets covering 693 cancer-related genes, seven promoter regions and selected fusions (Supplementary Table 1). NGS libraries were constructed following the manufacturer’s manual. Library preparation failed in eight cases. Sequencing was performed on NovaSeq6000 or NextSeq500 instruments (Illumina, San Diego, CA, USA) in the paired-end mode as specified by the manufacturer. Two samples had to be excluded because of barcode issues or sample contamination. Sequencing was performed for tumour and normal tissue in 210 cases.

### Data analysis

Raw data was processed using the megSAP data analysis pipeline (https://github.com/imgag/megSAP) combined with the ngs-bits package (https://github.com/imgag/ngs-bits). Briefly, sequencing reads were aligned to the human reference genome (GRCh38) with BWA-MEM, somatic variants were called using Strelka2 and annotated with Variant Effect Predictor (VEP) and cancer-specific databases such as COSMIC [19–21].

Quality control was performed using QC tools from the ngs-bits package [22]. Fifteen tumour (FFPE) samples failed to pass QC filters, with a median depth of less than 20x over at least 80% of the target regions. Two cases had to be excluded due to the detection of tumour DNA in normal samples. After quality control, 193 patients remained for further analysis (Supplementary Table 2). To obtain high-confidence variant lists, variants were required to have a tumour and normal depth-of-coverage of at least 20x, an allelic frequency of 5% or more and a minimum of 3 reads showing the alternative allele. Mutations in *TERT* promoter hotspots were analysed independently (see Supplementary Table 3).

All samples were manually assigned to one of four genomic subtypes described by TCGA: *BRAF, RAS, NF1* and Triple-WT [1]. Samples with hotspot mutations of the *BRAF* gene affecting the amino acids V600 or K601 were defined as *BRAF* subtype. If a mutation in one of the hotspot positions G12, G13 or Q61 was present in *NRAS*, *HRAS* or *KRAS*, the tumour was assigned to the RAS subtype. In case none of the mentioned mutations were present and an *NF1* mutation was detected, the sample belonged to the *NF1* subtype. Otherwise, it was classified as Triple-WT.

To detect candidate driver genes in the cohort, two versions of the intOGen pipeline were used [23]. The current intOGen version consists of seven individual tools and applies a statistical combination method on the individual results to report a unified list of candidates. The algorithms include recurrence (mutation count) based approaches (CBaSE, MutPanning, dNdScv), mutation clustering in protein context (OncodriveCLUSTL, HotMAPS, smRegions) and functional impact bias (OncodriveFML). A Q-value cut-off of 0.10 was used on the combination output. In addition, we generated a recurrence analysis with MutSigCV, a commonly used tool included in a previous version of intOGen (see Supplementary Table 4 for results) [24].

For SCNA detection, ClinCNV (version 1.16) was used [25]. By incorporating off-target reads, the tool can analyse CNVs genome-wide, even for targeted panel sequencing data. ClinCNV provides false discovery rates per CNV and per sample. Nine samples exceeded the per-sample FDR limit of 5% (QC threshold indicating low-quality SCNA analysis), preventing automated analysis with ClinCNV. Six of them could be manually analysed by specifying coordinates of diploid regions as input to ClinCNV based on a visual inspection of the results in Integrative Genomics Viewer (IGV) [26]. The remaining three had to be excluded from further SCNA analysis due to low data quality. Correction for purity and heterogeneity was performed to obtain integer copy numbers. A gene with an integer copy-number (CN) of ≥ 4 was defined as amplified. A heterozygous deletion was assumed with an integer CN = 1, a homozygous deletion when CN = 0.

Amplified or deleted regions in the cohort were analysed by GISTIC (version 2.0.23), an algorithm that searches for signals of positive selection of SCNAs [27, 28]. No cut-off for amplifications or deletions was used for this analysis since the algorithm needs passenger alterations to create a background model [28]. The algorithm corrects for multiple testing using the FDR method. The threshold of significance was set at 0.25. The impact on survival of the five recurrently deleted regions with the lowest q-values in GISTIC was analysed. GISTIC output was used as input for survival analysis with Kaplan-Meier curves and univariate and multivariate Cox proportional hazards models. Samples with an amplitude threshold of < −0.1 for the respective region were included to eliminate artefacts.

We used cBioPortal to extract somatic mutation (SNVs, indels and SCNA) counts and frequencies in the gene *CBL* observed in the previous studies (1) Skin Cutaneous Melanoma (TCGA, PanCancer Atlas), (2) Melanoma (MSKCC, Clin Cancer Res 2021), and 3) Melanoma (MSK, NEJM 2014), among other smaller melanoma studies (see Fig. 3b) [29–31]. Frequencies of observed somatic variants were visualized using cBioPortal’s OncoPrint function. The distribution of point mutations and hotspots in *CBL* was visualized using cBioPortal’s lollipop-plot function.

### Statistical analysis

For survival analysis, follow-up (FUP) time was defined as the time from primary diagnosis to the date of last FUP or death by any cause. Relapse-free survival was defined as the time between diagnosis of primary tumour and loco-regional or distant recurrence. RFS exclusively included the period during which patients did not receive any adjuvant therapy until recurrence. Overall survival (OS) was defined as the time between primary diagnosis and death by any cause. April 2019 was the cut-off date for data collection.

Kaplan-Meier curves and univariate and multivariate Cox proportional hazards models were used for statistical analysis. The Cox proportional hazards assumption was fulfilled for all analysed variables.

Statistical analysis and visualization were performed using R statistical language (version 4.1.1) with relevant packages (see supplement), the scipy Python package (version 1.11.4) and the lifelines Python package (version 0.27.8) [32–34]. *P*-values below 0.05 were considered statistically significant.

## Results

We screened 1163 stage II patients who were diagnosed between 2000 and 2018, as identified in the CMMR. Tissue blocks were available for 290 of these patients. For 31 samples, no tumour or no normal DNA was obtained from the available tissue block. In 39 cases, the DNA concentration was too low for sequencing. 10 cases had low sequencing quality, further 17 cases were removed because of low data quality. A total of 193 patients were included in this study. The majority of patients were male (61.1%), with a median age at diagnosis of 72 years (range: 24–92 years). Most patients (42.0%) were diagnosed with stage IIA disease, followed by 33.7% with stage IIB and 24.4% with stage IIC. The most common histology was SSM (36.8%), followed by NM (24.4%), ALM (17.1%), and LMM (10.4%).

### Molecular characterization

**Fig. 1:**
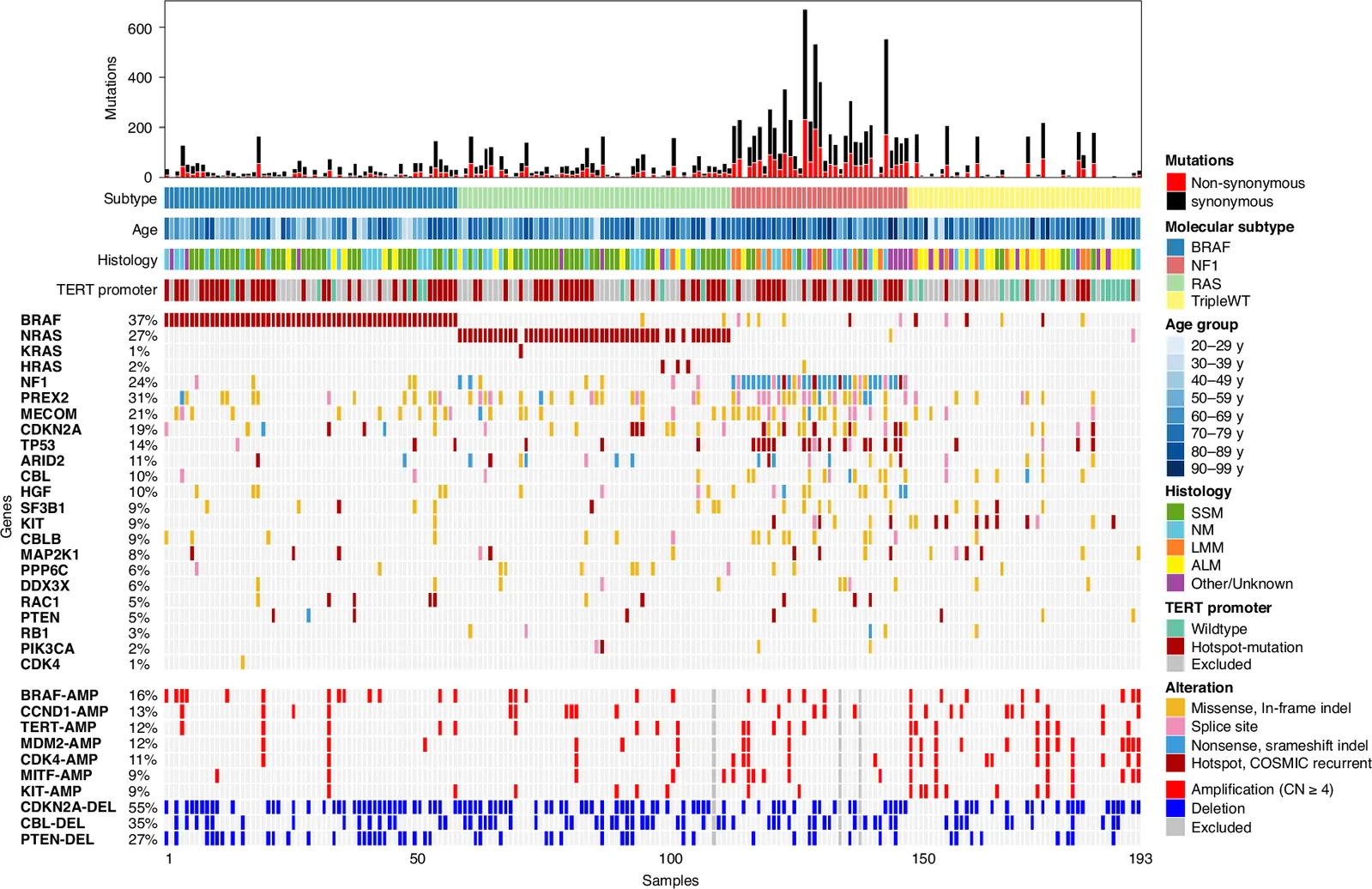
Genetic landscape of stage II melanoma. Oncoplot showing selected clinical and somatic features in stage II melanoma (*n* = 193). Samples are arranged on the x-axis by molecular subtype. Mutation count, molecular subtype, patient age and histology are shown in the upper part. Below, hotspot mutations in the TERT promoter as well as somatic mutations (middle), amplifications (CN > 4) and deletions of selected genes are shown. While most presented genes are known drivers in melanoma, we identified a putative novel driver gene, CBL, which shows a highly significant recurrence of deletions in all subtypes and a significant recurrence of point mutations in the NF1 subtype.

**Fig. 2:**
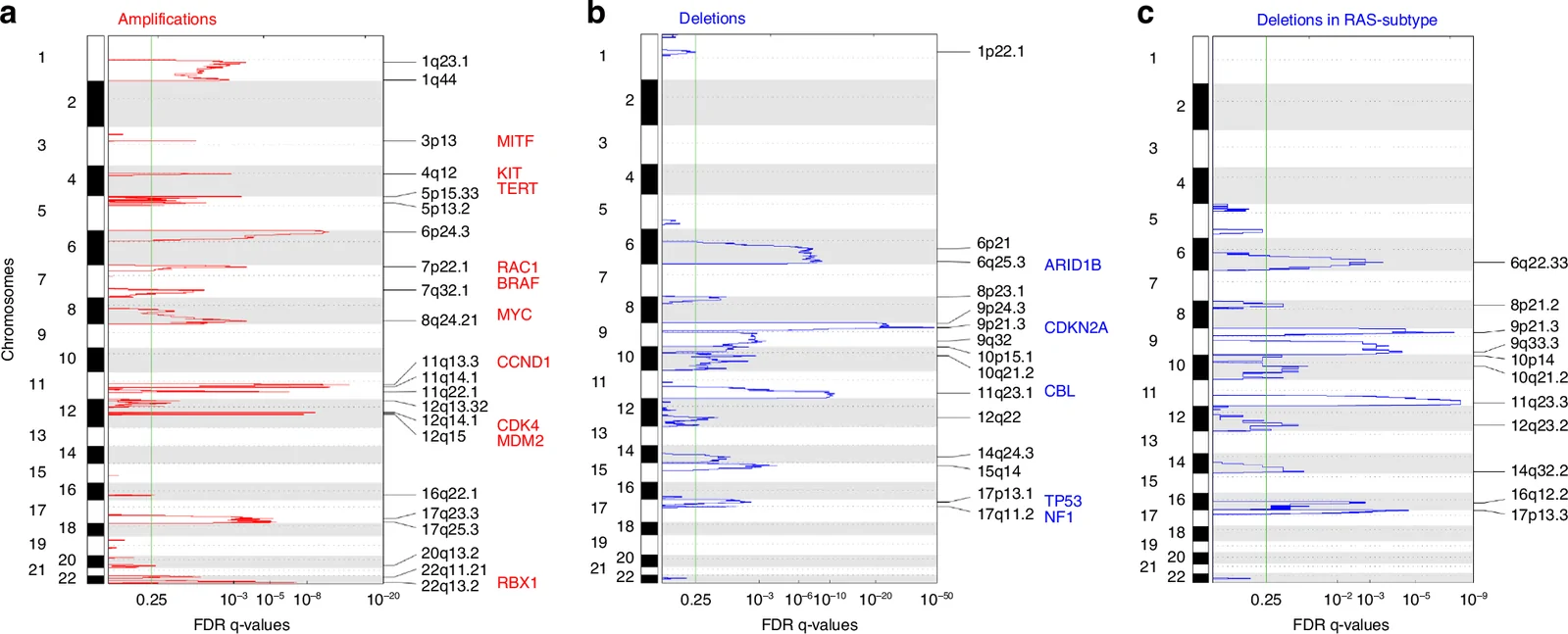
Significant copy-number changes. **a** Significantly amplified and **b** significantly deleted chromosomal regions detected in the cohort. **c** Significantly deleted chromosomal regions detected in RAS-subtype samples.

Figure 1 and 2 give an overview of the genetic landscape of this stage II melanoma cohort. Fifty-eight tumours (30.1%) were classified as *BRAF* mutated subtype with mutations affecting the amino acids V600 or K601. Non-hotspot mutations in the *BRAF* gene were detected in 17 tumours (Supplementary Table 5), one synonymous mutation and one in combination with the V600E *BRAF* hotspot mutation. 54 melanomas (28.0%) harboured mutations in the hotspots G12/G13 or Q61 of *NRAS*, *HRAS* or *KRAS* and were thus defined as RAS-positive. *BRAF* and *RAS* hotspot mutations occurred mutually exclusively (Fisher’s exact test: p = 5 × 10^−^^10^). Of the remaining patients, 35 tumours showed non-synonymous coding or splice-site mutations in the *NF1* gene (18.1%). Finally, 46 tumours were defined as Triple-WT (23.8%).

Numerous additional genes were impacted by mutations, deletions, or amplifications, many of which are frequently altered in more advanced stages of melanoma (Fig. 1). The *NF1* subtype, in particular, demonstrated a relatively high mutational burden, with frequently mutated genes including *TP53*, *PREX2* and *CDKN2A*. Furthermore, *NF1* patients were diagnosed at an older age compared to the rest of the cohort (Mann-Whitney U Test: p = 0.001). In contrast, Triple-WT melanomas exhibited a relatively low number of coding mutations and TERT promoter hotspot mutations. Despite this, the number of KIT mutations with COSMIC annotation was exceptionally high in Triple-WT tumours.

Genes affected by recurrent deletion in the whole cohort include *CDKN2A* (55% of all patients) and *PTEN* (27%), as well as the previously unreported gene *CBL* (35% of patients, Fig. 1 bottom). Genes affected by amplifications are less common, with *BRAF*, *CCND1*, *TERT*, *MDM2* and *CDK4* being the most common.

### Identification of potential driver genes

The driver detection methods MutSigCV, OncodriveFM and OncodriveClust (Supplementary Table 4) nominated 19 genes as potential driver genes. Seven of these genes were detected by all three algorithms: *BRAF*, *NRAS*, *CDKN2A*, *TP53*, *MAP2K1*, *PPP6C* and *CBL*. Among these, *CBL* represents a novel observation as a potential driver gene in cutaneous and acral melanoma, which has not been reported previously in the context of melanoma oncogenesis. Twelve genes passed FDR thresholds in at least two of three in-silico prediction tools. Those were *NF1*, *TET2*, *IL7R*, *ARID2*, *DICER1*, *SF3B1*, *HGF*, *NUP98*, *PTEN*, *CDC27*, *TCF12* and *RPL5*. All 19 genes also achieve a highly significant combined q-value using a combination of seven driver prediction methods implemented in the latest intOGen pipeline (see Methods). Mutation frequencies of identified genes can be found in the Supplementary Table 4.

This newly identified role of *CBL* as a driver gene in melanoma is supported by robust in-silico predictions from multiple algorithms (MutSigCV, OncodriveFM, and OncodriveClust), as well as by the identification of a highly significant hotspot in the zinc finger domain (positions 417 and 418, Supplementary Fig. 1b, c). Interestingly, *CBL* mutations are frequently found in NF1-type melanoma, with a significant enrichment of *CBL* alterations observed in two large melanoma studies (TCGA and MSK, Supplementary Table 10). These findings highlight a potential subtype-specific association, suggesting that CBL alterations may play a distinct role in the biology of *NF1*-mutated melanomas.

**Fig. 3:**
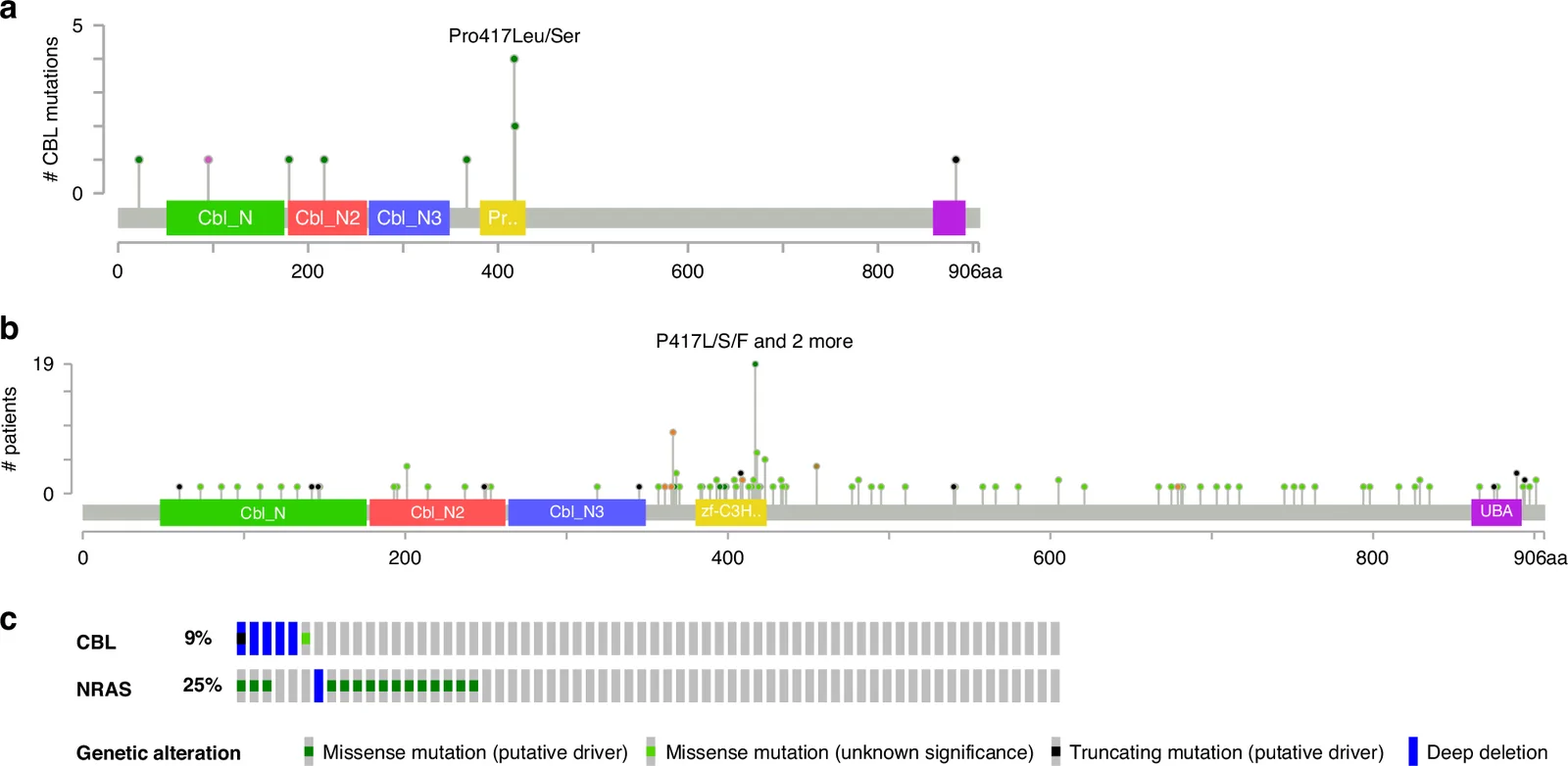
Mutation hotspot CBL-417/418 affecting the amino acids in positions 417 and 418. **a** 7 mutations in positions 417 and 418 identified in our study. **b** 25 mutations identified in positions 417 and 418 in cBioPortal (plus 5 mutations in position 423). **c** Three out of five patients in the MSK study affected by a deep deletion of CBL also carry a NRAS missense mutation.

We found 17 *CBL* missense mutations, two nonsense mutations and five splice site mutations across 20 tumours (Table 1). *CBL* mutations are found at a higher frequency in *NF1*-type melanoma (Supplementary Table 9, Fisher’s exact test *p* < 0.001), an enrichment we can replicate in two large melanoma studies from TCGA and MSK obtained from cBioPortal (Supplementary Table 10, Fisher’s exact test *p* = 0.032 in TCGA, *p* < 0.001 in MSK). The hotspot tools OncodriveCLUSTL, HotMAPS and smRegions identified a highly significant cluster of mutational activity at protein positions 417 and 418 (Fig. 3a). According to OncoKB [35], mutations in this *CBL* hotspot are classified as loss-of-function (LoF) or likely LoF. Notably, these *CBL* missense mutations are mutually exclusive with *BRAF* or *RAS* hotspot mutations (Fisher's exact test: *p* = 0.0019 and *p* = 0.044) and are highly enriched in the *NF1*-mutated melanoma (6 of 7 *CBL* hotspot mutations).

**Table 1:** CBL mutations detected in the analysed stage II cohort.

PID	Variant	SCNA	Subtype	COSMIC count	Biological effect*
33	S22L	no	NF1	0	Unknown
12	R180W	het. del.	NF1	4	Unknown
73	R180W	no	Triple-wt	4	Unknown
3	S217F	no	NF1	0	Unknown
55	Q358*	no	Triple-wt	0	Unknown
59	V363K	no	Triple-wt	0	Unknown
65	Q367H	no	Triple-wt	1	Likely LoF [29]
61	Y371H	no	Triple-wt	28	LoF [30]
36	Y371N	no	NF1	3	Likely LoF [30]
45	P417L	het. del.	NF1	10	Likely LoF [31]
46	P417F	het. del.	NF1	10	Likely LoF [31]
	P417S			8	Likely LoF [31]
	c.2036+7 C > T (splice_region & intron)				Unknown
37^1^	P417S	het. del.	NF1	8	Likely LoF [31]
	F418I			3	Unknown
22	F418I	het. del.	NF1	3	Unknown
70	F418C	no	Triple-wt	0	Unknown
	c.590+1 G > C (splice_donor)				Unknown
28^2^	P582S	no	NF1	0	Unknown
8	E696K	no	RAS	0	Unknown
17	Q882*	no	NF1	0	Unknown
189	c.591-6 C > T (splice_region & intron)	amplification	BRAF		Unknown
154	c.2153+3 A > G (splice_region & intron)	het. del.	RAS		Unknown
24	c.2034 C > T/A678= (splice_region & synonymous)	no	BRAF		Unknown

*PID* patient identifier.

*according to OncoKB [32].

het. del. = heterozygous deletion.

amplification: integer copy-number ≥ 4.

^1^ = amelanotic, focally ulcerated, spindle-shaped, partially desmoplastic malignant melanoma.

^2^ = desmoplastic malignant melanoma.

We could replicate this novel hotspot *CBL* 417/418 in skin cutaneous melanoma studies provided in cBioPortal (cbioportal.org) with 19 and 6 patients showing an amino-acid change in positions 417 and 418, respectively. Another five patients are affected by a change in the nearby amino acid at position 423, resulting in a total of 30 patients affected in this *CBL* hotspot (Fig. 3b). Moreover, the zf-C3HC4_3 zinc finger domain harbouring this hotspot shows an enrichment of mutations across the whole domain, including its upstream and downstream region, with more than 50% of all variants in CBL clustering around zf-C3HC4. Interestingly, the remaining mutations outside the zinc-finger domain are highly enriched for truncating mutations (Fig. 3a). Point mutations in *CBL* are also enriched for the *NF1*-subtype in the TCGA-Melanoma and MSK-2021-Melanoma cohort available on cBioPortal, with 14.8% and 30.2% affected patients, respectively (Supplementary Table 10).

### Driver genes in copy number alterations

Figure 2 shows significantly amplified (panel A) and deleted (panel B) chromosomal regions detected by GISTIC. Among others, the genes *CDKN2A*, *KIT*, *MITF*, *TERT*, *BRAF*, *CCND1*, *CDK4*, *MDM2* and *MYC* were located in significant regions. As expected, regions harbouring oncogenes were affected by amplifications, those with tumour suppressor genes by deletions. Peaks of the five deleted regions with the lowest q-values were at 9p21.3, 9p24.3, 11q23.1-3, 6q25.3 and 6q21. The chromosomal start and end points of these regions can be found in Supplementary Table 6. Region 11q23.1-3 contained more than 250 genes (Supplementary Table 7) in its wide-peak, among others *CBL*. 14 of these genes are annotated as oncogenes or tumour suppressor genes in the Network of Cancer Genes or Cancer Gene Census (Supplementary Table 8), however, of these, only *CBL* was also significant in the driver gene analysis described above.

In total, 83 of the 193 patients (43.0%) were identified as being affected by a deletion of this region (GISTIC FDR *q* < 10^−^^10^, Fig. 2b). With a median of 75 years (IQR: 63.0–80.5 years) 11q23.1-3 deletions were significantly associated with higher patient age compared to a median of 69.5 years (IQR: 57.75–79.25 years) in wild-type patients (Mann–Whitney U test: *p* = 0.02). There were no significant differences between gender (Fisher’s exact test: *p* = 0.45), AJCC stage at diagnosis (Chi-squared test: *p* = 0.55) and histology (Chi-squared test: *p* = 0.62).

The 11q23.1-3 deletion shows strong recurrence in the RAS-subtype (Fig. 2c, Supplementary Table 9), in which this deletion shows a stronger significance than the highly frequent *CDKN2A* deletion. The narrow peak of the recurrent deletion (region deleted in all affected patients) in the RAS-subtype (Fig. 2c) specifically spans only the 11q23.3 region, which contains the *CBL* gene amongst others, further reinforcing our conclusion that *CBL* is an essential driver gene.

The 11q23.1-3 deletion is less frequent in the TCGA Skin Cutaneous Melanoma [29] and the MSK- Melanoma [31] studies, for which GISTIC results are available on cBioPortal. TCGA reported 3% of patients, while MSK reported 8% of patients affected by a deep deletion of *CBL*, which might explain why the deletion has not been reported as a driver event in melanoma yet. Interestingly, 60% of patients affected by this deletion in the MSK study also carry an *NRAS* mutation (Fig. 3c), showing a similar enrichment in that subtype as observed in our study.

### Survival analyses

The median follow-up time was 27 months (IQR 47.5). 54 events were recorded for RFS and in case of relapse, the median RFS time was 14 months (IQR 13). Thirty-two patients had a relapse classified as stage IV, 22 were classified as stage III. For OS, there were 41 events with a median OS time of 26 months (IQR 27). A relapse was documented for 26 of 41 patients who died during follow-up.

**Fig. 4:**
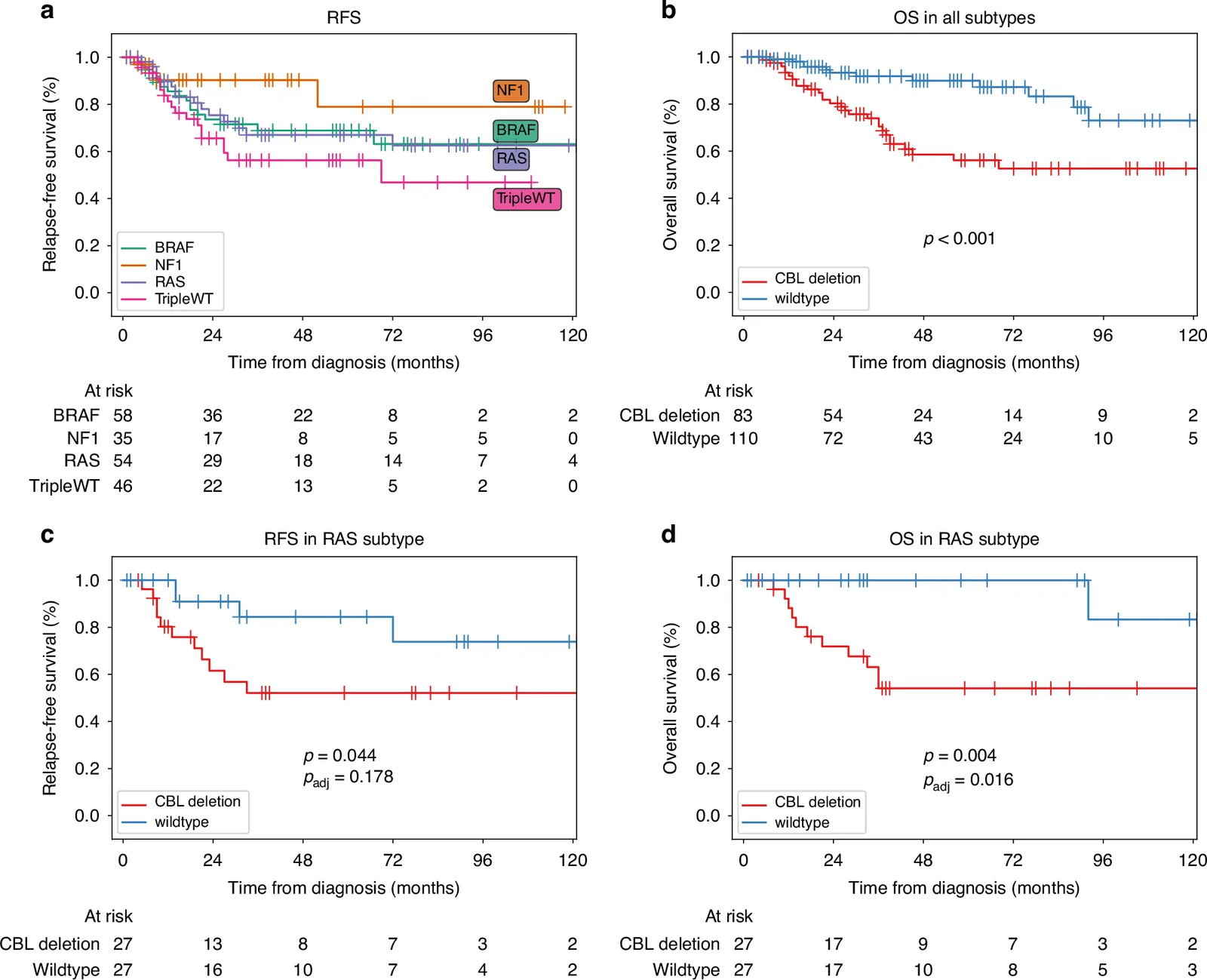
Impact of molecular subtype and CBL-deletion status on survival. **a** Triple-WT shows a significantly worse relapse-free survival (RFS) compared to the BRAF subtype. **b** CBL-deletion (11q23.1-3) carriers show a significantly worse overall survival (OS) in all melanoma subtypes. **c** CBL-deletion carriers with the RAS subtype show significantly worse RFS compared to non-carriers. **d** CBL-deletion carriers with the RAS subtype show significantly worse OS compared to non-carriers.

Analysis of molecular subtypes found the *NF1* subtype to be associated with a significantly better RFS compared to Triple-WT patients (*p* = 0.03, HR = 0.30, CI: 0.10–0.89). The association remained significant in our multivariate analysis after correction for age and stage. Kaplan-Meier analysis supports the significantly better RFS of the *NF1* subtype compared to Triple-WT (Fig. 4a). Concerning OS, we observed a strong and nearly significant (*p* = 0.06) reduction in hazard ratio (HR = 0.43, CI: 0.18–1.04) for *BRAF* patients. Similarly, Kaplan-Meier analysis shows improved (but not significantly improved) OS for the *BRAF* subtype (Supplementary Fig. 2), warranting further investigation in future studies.

Next, we tested for differences in outcome associated with deletions of the five genomic regions showing the smallest q-values in GISTIC analysis (Supplementary Table 6), namely 9p21.3, 6q25.3, 11q23.1-3, 6q22.1 and 10q26.13. Only for the region 11q23.1-3, we found a significant difference in OS between deletion and wildtype samples in Kaplan-Meier analysis (Fig. 4b). Patients whose tumours harboured a deletion of the region showed worse OS (HR = 3.40, 95% CI = 1.73–6.69, *p* < 0.001). This association remained significant in multivariate Cox-analysis (HR = 2.31, CI: 1.13–4.73, *p* = 0.022) after correction for patients’ age, tumour stage and molecular subtype. However, the effect of the 11q23.1-3 deletion on RFS was not significant in the whole cohort (Supplementary Fig. 3a).

As the deletion shows the highest recurrence in the *RAS* subtype, we performed Kaplan-Meier RFS and OS analysis for this subgroup. We found that patients harbouring the 11q23.1-3 deletion and *RAS* mutations have significantly reduced RFS (*p* = 0.044, Fig. 4c) and OS (*p* = 0.004, Fig. 4d), which remained significant following Benjamini-Hochberg correction, while no significant changes of RFS or OS could be observed for other subtypes (Supplementary Fig. 3b–d). Hence, our study revealed the 11q23.1-3 deletion harbouring the gene *CBL* as a prognostic biomarker in the *RAS* subtype.

## Discussion

The reported genetic landscape of stage II melanoma revealed known drivers, a novel driver gene, *CBL*, and a potential prognostic marker for relapse-free and overall survival, namely the deletions in the region 11q23.1-3, which includes the gene *CBL*.

The gene *CBL* is of special interest since it was detected as a potential driver in our cohort by multiple algorithms. The gene had been described as a driver of desmoplastic melanoma before [36]. However, in our cohort, only two (10%) of the *CBL*-mutated tumours had desmoplastic or partially desmoplastic histology (Table 1), which implies that the gene may play a role in other histological subtypes. Indeed, we identified a hotspot mutation at protein positions 417–418, which affects a zinc finger domain, is highly enriched in the NF1-subtype of melanoma, and could be replicated in previous studies provided on cBioPortal. *CBL* encodes an E3 ubiquitin-protein ligase [37]. E3 ubiquitin-protein ligases contribute to the degradation of activated receptor tyrosine-kinases (RTK) and, as a consequence, they can downregulate cellular signalling pathways like MAPK- and PI3K-signalling [38–40]. Furthermore, indirect inhibition of the MAPK pathway was assumed through the binding of CBL to Grb2 competitively to SOS [41].

Shain and colleagues assumed a tumour-suppressive function for *CBL* in desmoplastic melanoma because the gene was affected by a high fraction of truncating missense variants, an effect we also observed in cases provided on cBioPortal (Supplementary Fig. 1a) [36]. The detection of two mutational hotspots containing variants, which were classified as loss-of-function (LoF) or likely LoF by OncoKB (Table 1, https://www.oncokb.org/). *CBL* was considered a suppressor gene in lung cancer and *CBL* status is a potential positive indicator for MET-targeted therapeutics in NSCLC [42].

However, the role of *CBL* is complex and cell-context dependent. Nihal and Wood showed *CBL* to be highly expressed in melanoma cell lines and siRNA knockdown of CBL reduced oncogenicity [43]. However, four of the cell lines were known to carry activating *BRAF* mutations in the MAPK-signalling downstream of RTKs (depmap.org) and likely render tumour-suppressive *CBL* function irrelevant in this context. As described before, *CBL* hotspot mutations were found to be mutually exclusive with activating *BRAF* and *RAS* mutations in our cohort [36]. This supports the hypothesis that *CBL* contributes to the activation of MAPK-signalling in cutaneous malignant melanoma.

Besides *CBL*, we were able to confirm known driver genes in early-stage melanoma. This was particularly the case for genes with small q-values reported by driver gene predictors, like *BRAF*, *NRAS*, *NF1*, *CDKN2A*, *PTEN* and *TP53* [2–5, 44–46]. We were able to confirm recently identified driver genes like *MAP2K1*, *PPP6C* and *ARID2* [2, 6, 7]. *SF3B1* had already been described in mucosal and uveal melanoma [47, 48]. GISTIC analysis detected significantly altered chromosomal regions containing known melanoma drivers. Prominent examples are amplifications of *KIT*, *MITF*, *TERT*, *BRAF*, *CCND1*, *CDK4*, *MDM2* and *MYC* as well as deletions of *CDKN2A* [1, 2, 49–54]. These results indicate that most of the important melanoma drivers are already affected at an early stage. Especially, Triple-WT tumours were found to have high numbers of focal amplifications as already described by TCGA [1]. SCNAs could therefore act as an alternative mechanism of oncogenic activation in Triple-WT tumours. However, it is still possible that the frequency of SCNA-affected tumours increases during progression to higher stages.

Age, AJCC stage and tumour thickness were confirmed as prognostic markers in the analysed cohort [18, 55]. In contrast, the established prognostic markers, gender and ulceration, did not show a significant effect on survival.

The prognostic value of mutations in *BRAF*, *NRAS* or *NF1* has been controversial [1, 13–15, 56]. In a recent study, the authors found an association of *BRAF* and *NRAS* mutations with worse melanoma-specific survival (MSS) in primary tumours of at least stage IIB [15]. A second study reported worse MSS and OS for *NF1*-mutated melanoma, but no significant difference for primary tumours [16]. Krauthammer and colleagues, however, did not find significant differences in OS dependent on *NF1*, *BRAF* or *RAS* mutations [57]. We did not observe significant differences in OS and RFS for *BRAF* and *RAS* subtypes as well. However, there was a trend for better OS of *BRAF*-mutated melanoma. RFS was improved for *NF1*-mutated tumours. It could be speculated that a higher mutation burden associated with the *NF1* subtype leads to better immune control [58]. Although point mutations in *CBL* were identified as potential driver events, no association with RFS or OS was found.

Deletions of a genomic region with its peak at 11q23.1-3 were significantly associated with worse OS in our study. Deletions of 11q23 in melanoma were already described to be associated with regional lymph node metastases [59]. The authors observed loss of heterozygosity (LOH) of the region in 38% of primary tumours, in 67% of regional lymph node metastases and in 100% of distant metastases [59]. Even though the number of studied tumours was small, this supports our findings and could hint at an association with aggressive tumour behaviour and the tendency to metastasize. We observed the strongest effect of deletions, highly significantly worse RFS and OS, in the RAS-subtype, in which 11q23.1-3 is also most frequent, making this mutation a potent prognostic biomarker for patients with RAS mutations. Although 11q23.1-3 was also highly recurrent in *NF1* and Triple-WT (but less frequent in *BRAF*-mutated), we did not observe a significant effect on survival in these subtypes. Interestingly, hotspot point mutations in *CBL* are highly enriched in *NF1* and mutually exclusive with RAS mutations, possibly hinting at different consequences of *CBL* hotspot mutations and *CBL* deletions. Changes in the expression of *CBL* have already been reported to influence survival in other tumour entities. High *CBL* expression was described to be associated with improved OS in stage IV colorectal cancer and reduced progression-free survival and OS in glioma and skull base chordoma [60–62]. However, because the region detected by GISTIC affects a higher number of genes, it is not clear whether the association is due to the deletion of *CBL* or the combination of affected genes. Narrow-peak analysis in the RAS subgroup, however, supports the essential role of *CBL*, as this gene is included in the intersection of all 11q23.1-3 deletions in RAS patients that only spans 11q23.3.

Our findings support the prognostic role of known melanoma driver genes and identify *CBL* as a potential new driver in cutaneous melanoma. Deletions of 11q23.1-3, including CBL, were identified as a prognostic marker for worse survival in our cohort of stage-II-melanoma patients, as well as for worse RFS and OS in patients with RAS mutations. These deletions may be considered a potential prognostic biomarker that could be used to guide patients’ inclusion in future studies. Still, additional investigations are needed to define the functional relevance of CBL.

## Supplementary information

Supplementary Material

## Data Availability

The datasets used and analysed during the current study are available from the corresponding author on reasonable request.
