# Supplementary material for: Genetic landscape of stage II melanoma identifies CBL as a new driver gene and prognostic biomarker
Source: Br J Cancer. 2026 Apr 9;134(12):1801–9. doi: 10.1038/s41416-026-03394-1 (PMC13226680; doi:10.1038/s41416-026-03394-1)

**Supplementary Methods / Material for**

“*Genetic landscape of stage II melanoma identifies CBL as a new driver gene and prognostic biomarker”*

**Table of Contents**

[Supplementary Methods: 2](#_Toc220317560)

[TERT Promoter Hotspot mutations 2](#_Toc220317561)

[R packages used for figures and statistical evaluation 2](#_Toc220317562)

[Supplementary Tables 3](#_Toc220317563)

[Table 1: Sequenced genes. 3](#_Toc220317564)

[Table 2: Sequencing quality of included patient's samples. 5](#_Toc220317565)

[Table 3: Sequenced promoter regions. 5](#_Toc220317566)

[Table 4: Genes with significant q-values in at least two of three driver-gene prediction algorithms MutSigCV, OncodriveClust and OncodriveFM and a significant combined q-value of the seven driver-prediction tools included in the latest version of intOGen pipeline (see Methods). The second column shows the fraction of samples harboring a point mutation (somatic SNVs or indels) in the respective genes. 5](#_Toc220317567)

[Table 5: BRAF mutations not affecting V600/K601 hotspot. 6](#_Toc220317568)

[Table 6: Limits of top five deleted regions detected by GISTIC. 7](#_Toc220317569)

[Table 7: Genes located on the wide-peak of region 11q23.1-3 detected by GISTIC. 7](#_Toc220317570)

[Table 8: Potential cancer driver genes located on the wide-peak of region 11q23.1-3 detected by GISTIC. 9](#_Toc220317571)

[Table 9: Somatic mutations in CBL: number of patients affected SNVs or CNVs in CBL. 10](#_Toc220317572)

[Table 10: Somatic SNVs in *CBL* in TCGA and MSK cohorts. Patients affected by SNV and CNV in *CBL* were enriched in NF1 subtype (highlighted in the table). 11](#_Toc220317573)

[Table 11: Cancer driver gene prediction using 7 methods included in the intOGen pipeline 12](#_Toc220317574)

[Supplementary Figures 15](#_Toc220317575)

[Supplementary Figure 1: (A) Driver variants in CBL identified in cBioPortal cluster in a zinc finger domain. (B) CBL harbours mutations in ˜9% of patients in 9 studies included in cBioPortal. (C) In TCGA PanCancerAtlas, the frequency of CBL mutations is ~7%. 15](#_Toc220317576)

[Supplementary Figure 2: Improved OS of BRAF-subtype compared to Triple-WT (not significant, p=0.06). 16](#_Toc220317577)

[Supplementary Figure 3: The effect of the CBL deletion on RFS is not significant across the whole cohort and in the *BRAF*, *NF1* and Triple-WT molecular subtypes. 17](#_Toc220317578)

## Supplementary Methods:

### TERT Promoter Hotspot mutations

Since the coverage of the TERT promoter region was not sufficient in all samples, TERT hotspots were manually analyzed for the presence of mutations and coverage of the position. Samples with a read depth of at least 20x in all hotspot locations (n=65) were regarded as having good quality and were therefore included in the analysis. If the depth of one or more of the five hotspots was between 10 and 20x, the decision about the inclusion was made individually. When in the case mentioned above, a mutation with at least three reads and 5 % allelic frequency was detected in the tumor, the sample was included. If no such variant was found or when the depth was below 10x, the sample was excluded from further analysis. This approach led to the inclusion of 111 samples for further analysis.

### R packages used for figures and statistical evaluation

Analyses were conducted in R (version 4.1.1) using the following packages: tidyverse (v1.3.1), survival (v3.2), survminer (v0.4.9), caret (v6.0), leaps (v3.1) and openxlsx (v4.2.4).

## Supplementary Tables

Table 1: Sequenced genes.

| *ABCB1* | *CD274* | *EIF4A2* | *GNAI1* | *LRRK2* | *NSD1* | *RAD51D* | *STK19* |
| --- | --- | --- | --- | --- | --- | --- | --- |
| *ABCC2* | *CD276* | *ELF3* | *GNAQ* | *LYN* | *NSD2* | *RAD52* | *STK31* |
| *ABCC4* | *CD70* | *EML4* | *GNAS* | *LZTR1* | *NSD3* | *RAD54L* | *STK40* |
| *ABCG2* | *CD79A* | *EP300* | *GOLGA5* | *MAD2L2* | *NTN4* | *RAF1* | *SUFU* |
| *ABL1* | *CD79B* | *EPCAM* | *GOT1* | *MALAT1* | *NTRK1* | *RANBP2* | *SULT1A1* |
| *ABL2* | *CDC27* | *EPHA2* | *GPS2* | *MAML2* | *NTRK2* | *RARA* | *SUZ12* |
| *ABRAXAS1* | *CDC73* | *EPHA3* | *GPX1* | *MAP2K1* | *NTRK3* | *RASA1* | *SYK* |
| *ACO1* | *CDH1* | *EPHA5* | *GREM1* | *MAP2K2* | *NUMA1* | *RB1* | *SYNE1* |
| *ACTB* | *CDH20* | *EPHA6* | *GRIN2A* | *MAP2K4* | *NUP93* | *RBM10* | *TAF1* |
| *ACVR1B* | *CDK12* | *EPHA7* | *GRM3* | *MAP2K7* | *NUP98* | *RBMX* | *TAP1* |
| *ACVR2A* | *CDK2* | *EPHB1* | *GSK3B* | *MAP3K1* | *NUTM1* | *RECQL4* | *TBL1XR1* |
| *ADAM10* | *CDK4* | *EPHB2* | *GSTP1* | *MAP3K13* | *OR5A1* | *REL* | *TBX3* |
| *ADAMTS18* | *CDK6* | *EPHB6* | *GUCY1A2* | *MAP3K3* | *OTC* | *RET* | *TCF12* |
| *ADCY1* | *CDK8* | *EPPK1* | *GUSB* | *MAP3K4* | *PABPC1* | *RHBDF2* | *TCF3* |
| *ADGRA2* | *CDKN1A* | *ERBB2* | *H3F3A* | *MAP4K1* | *PAK1* | *RHEB* | *TCF7L2* |
| *AJUBA* | *CDKN1B* | *ERBB3* | *H3F3C* | *MAP4K3* | *PAK3* | *RHOA* | *TERT* |
| *AKAP9* | *CDKN2A* | *ERBB4* | *HCFC1* | *MAPK1* | *PAK5* | *RICTOR* | *TET1* |
| *AKT1* | *CDKN2B* | *ERCC1* | *HERC1* | *MAPK8IP1* | *PALB2* | *RIT1* | *TET2* |
| *AKT2* | *CDKN2C* | *ERCC2* | *HGF* | *MAX* | *PARP1* | *RNF43* | *TFDP1* |
| *AKT3* | *CEBPA* | *ERCC3* | *HIF1A* | *MBD1* | *PARP2* | *ROS1* | *TGFBR1* |
| *ALK* | *CHD1* | *ERCC4* | *HIST1H1C* | *MC1R* | *PARP3* | *RPA1* | *TGFBR2* |
| *ALOX12B* | *CHD3* | *ERCC5* | *HIST1H2BD* | *MCL1* | *PARP4* | *RPGR* | *TGIF1* |
| *AMER1* | *CHD4* | *ERG* | *HIST1H3B* | *MDC1* | *PAX5* | *RPL22* | *TIPARP* |
| *ANK3* | *CHD8* | *ESR1* | *HLA-A* | *MDM2* | *PAX8* | *RPL5* | *TJP2* |
| *APAF1* | *CHEK1* | *ESR2* | *HLA-B* | *MDM4* | *PBRM1* | *RPS15* | *TLR4* |
| *APC* | *CHEK2* | *ETV1* | *HNF1A* | *MECOM* | *PCBP1* | *RPS6KA4* | *TMEM127* |
| *APCDD1* | *CHUK* | *ETV4* | *HOXD8* | *MED1* | *PCSK9* | *RPS6KB2* | *TMEM43* |
| *AR* | *CIC* | *ETV5* | *HRAS* | *MED12* | *PDCD1* | *RPTOR* | *TMPRSS2* |
| *ARAF* | *CLTC* | *ETV6* | *HSD3B1* | *MED17* | *PDGFRA* | *RUNX1* | *TNF* |
| *ARFRP1* | *CNOT1* | *EWSR1* | *HSP90AB1* | *MED23* | *PDGFRB* | *RUNX1T1* | *TNFAIP3* |
| *ARHGAP35* | *CNTNAP1* | *EXT1* | *HSPA8* | *MEF2A* | *PDK1* | *RUNX3* | *TNFRSF14* |
| *ARHGEF6* | *COL1A1* | *EXT2* | *ICOSLG* | *MEF2B* | *PDPK1* | *RXRA* | *TNNI3* |
| *ARID1A* | *COL3A1* | *EZH1* | *IDH1* | *MEN1* | *PHF6* | *RYBP* | *TNNT2* |
| *ARID1B* | *COMT* | *EZH2* | *IDH2* | *MET* | *PHOX2B* | *RYR1* | *TNPO1* |
| *ARID2* | *COP1* | *FAM46C* | *IFNGR1* | *MGA* | *PIK3C2B* | *SACS* | *TOM1* |
| *ARID5B* | *CRBN* | *FANCA* | *IGF1* | *MITF* | *PIK3C2G* | *SAV1* | *TOP1* |
| *ASXL1* | *CREB1* | *FANCC* | *IGF1R* | *MLH1* | *PIK3C3* | *SBDS* | *TOP2A* |
| *ASXL2* | *CREBBP* | *FANCD2* | *IGF2* | *MLH3* | *PIK3CA* | *SCN11A* | *TP53* |
| *ATM* | *CRKL* | *FANCE* | *IGF2R* | *MLLT10* | *PIK3CB* | *SCN5A* | *TP53BP1* |
| *ATP1A1* | *CRLF2* | *FANCF* | *IKBKE* | *MLLT3* | *PIK3CD* | *SDHA* | *TP63* |
| *ATR* | *CRTC1* | *FANCG* | *IKZF1* | *MMP2* | *PIK3CG* | *SDHAF2* | *TPMT* |
| *ATRX* | *CRTC3* | *FANCI* | *IL10* | *MN1* | *PIK3R1* | *SDHB* | *TPX2* |
| *AURKA* | *CSDE1* | *FANCL* | *IL7R* | *MNDA* | *PIK3R2* | *SDHC* | *TRAF3* |
| *AURKB* | *CSF1R* | *FANCM* | *ING1* | *MPL* | *PIM1* | *SDHD* | *TRAF7* |
| *AXIN1* | *CTCF* | *FAS* | *INHBA* | *MPO* | *PIP5K1A* | *SETBP1* | *TRIM28* |
| *AXIN2* | *CTLA4* | *FAT1* | *INPP4B* | *MRE11* | *PKP2* | *SETD2* | *TRIO* |
| *AXL* | *CTNNA1* | *FAT3* | *INPPL1* | *MSH2* | *PLCG1* | *SETDB1* | *TRRAP* |
| *B2M* | *CTNNB1* | *FBN1* | *INSR* | *MSH6* | *PLCG2* | *SF3B1* | *TSC1* |
| *BACH1* | *CTNND1* | *FBXO11* | *IRF4* | *MTHFR* | *PLK2* | *SGK1* | *TSC2* |
| *BAP1* | *CTTN* | *FBXW7* | *IRS1* | *MTOR* | *PMAIP1* | *SH2B3* | *TSHR* |
| *BARD1* | *CUL1* | *FES* | *IRS2* | *MUTYH* | *PML* | *SH2D1A* | *TTK* |
| *BBC3* | *CUL4A* | *FGF10* | *IRS4* | *MYB* | *PMS1* | *SHQ1* | *TXNIP* |
| *BCL2* | *CUL4B* | *FGF12* | *JAG1* | *MYBPC3* | *PMS2* | *SIN3A* | *TYMS* |
| *BCL2L1* | *CUX1* | *FGF14* | *JAG2* | *MYC* | *PNRC1* | *SLC19A1* | *U2AF1* |
| *BCL2L11* | *CYLD* | *FGF19* | *JAK1* | *MYCL* | *POLD1* | *SLC22A2* | *UBE2T* |
| *BCL2L2* | *CYP17A1* | *FGF23* | *JAK2* | *MYCN* | *POLE* | *SLC26A3* | *UBR5* |
| *BCL6* | *CYP1B1* | *FGF3* | *JAK3* | *MYD88* | *POLQ* | *SLCO1B3* | *UGT1A1* |
| *BCLAF1* | *CYP2C19* | *FGF4* | *JUN* | *MYH7* | *POLR3B* | *SLX4* | *UPF3B* |
| *BCOR* | *CYP2C8* | *FGF6* | *KALRN* | *MYH9* | *POU2F2* | *SMAD2* | *USP9X* |
| *BCORL1* | *CYP2C9* | *FGF7* | *KAT6A* | *MYL2* | *PPM1D* | *SMAD3* | *VHL* |
| *BCR* | *CYP2D6* | *FGFBP1* | *KCNH2* | *MYL3* | *PPP2R1A* | *SMAD4* | *VTCN1* |
| *BLM* | *CYP3A4* | *FGFR1* | *KCNQ1* | *MYLK* | *PPP6C* | *SMARCA1* | *WASF3* |
| *BMPR1A* | *CYP3A5* | *FGFR2* | *KDM5A* | *MYOD1* | *PRDM1* | *SMARCA4* | *WISP3* |
| *BRAF* | *DAXX* | *FGFR3* | *KDM5C* | *NAV3* | *PREX2* | *SMARCB1* | *WNK1* |
| *BRCA1* | *DCUN1D1* | *FGFR4* | *KDM6A* | *NBN* | *PRKAG2* | *SMARCD1* | *WRN* |
| *BRCA2* | *DDB2* | *FH* | *KDR* | *NCOA3* | *PRKAR1A* | *SMC1A* | *WT1* |
| *BRD4* | *DDR1* | *FLCN* | *KEAP1* | *NCOR1* | *PRKDC* | *SMC3* | *XIAP* |
| *BRIP1* | *DDR2* | *FLI1* | *KEL* | *NCOR2* | *PRKN* | *SMO* | *XPA* |
| *BTG1* | *DDX3X* | *FLT1* | *KIT* | *NEDD4L* | *PRPF8* | *SNCAIP* | *XPC* |
| *BTK* | *DDX5* | *FLT3* | *KLF4* | *NF1* | *PRSS8* | *SOCS1* | *XPO1* |
| *BUB1B* | *DIAPH1* | *FLT4* | *KLF5* | *NF2* | *PRX* | *SOD2* | *XRCC2* |
| *CAD* | *DICER1* | *FN1* | *KLHL6* | *NFE2L2* | *PSIP1* | *SOS1* | *XRCC3* |
| *CALR* | *DIDO1* | *FOXA1* | *KMT2A* | *NFKB1* | *PTCH1* | *SOX10* | *YAP1* |
| *CARD11* | *DIS3* | *FOXA2* | *KMT2B* | *NFKB2* | *PTEN* | *SOX17* | *YES1* |
| *CARM1* | *DMD* | *FOXE1* | *KMT2C* | *NFKBIA* | *PTGS2* | *SOX2* | *ZFHX3* |
| *CASP8* | *DNMT1* | *FOXL2* | *KMT2D* | *NIN* | *PTPN11* | *SOX9* | *ZFP36L1* |
| *CAST* | *DNMT3A* | *FOXP1* | *KRAS* | *NIPBL* | *PTPRC* | *SPEN* | *ZFP36L2* |
| *CBFB* | *DNMT3B* | *FOXQ1* | *LAMA2* | *NKX2-1* | *PTPRD* | *SPOP* | *ZMYM2* |
| *CBL* | *DOT1L* | *FUBP1* | *LATS1* | *NKX3-1* | *PTPRS* | *SPOPL* | *ZMYM3* |
| *CBLB* | *DPYD* | *GABRA6* | *LATS2* | *NOTCH1* | *PTPRT* | *SPTA1* | *ZNF217* |
| *CBLC* | *E2F3* | *GATA1* | *LCP1* | *NOTCH2* | *QKI* | *SPTAN1* | *ZNF703* |
| *CCAR1* | *EED* | *GATA2* | *LDLR* | *NOTCH3* | *RAC1* | *SRC* | *ZNF750* |
| *CCND1* | *EEF1A1* | *GATA3* | *LIFR* | *NOTCH4* | *RAD21* | *SRSF2* | *ZNF814* |
| *CCND2* | *EGFL7* | *GID4* | *LMNA* | *NPM1* | *RAD50* | *STAG2* | *ZRSR2* |
| *CCND3* | *EGFR* | *GLA* | *LMO1* | *NQO1* | *RAD51* | *STAT3* |  |
| *CCNE1* | *EGR3* | *GNA11* | *LPP* | *NR4A2* | *RAD51B* | *STAT4* |  |
| *CD1D* | *EIF1AX* | *GNA13* | *LRP1B* | *NRAS* | *RAD51C* | *STK11* |  |

Table 2: Sequencing quality of included patient's samples.

|  | **Median target region read depth** | **Median 100x percentage** |
| --- | --- | --- |
| Tumour tissue | 604x | 86,7% |
| Normal tissue | 518x | 82,9% |

Table 3: Sequenced promoter regions.

| **Chromosome** | **Start position** | **End position** | **Gene** |
| --- | --- | --- | --- |
| chr2 | 47629807 | 47630406 | *MSH2* |
| chr2 | 47636849 | 47637448 | *MSH2* |
| chr2 | 48009785 | 48010384 | *MSH6* |
| chr3 | 37034479 | 37035078 | *MLH1* |
| chr3 | 37034804 | 37035403 | *MLH1* |
| chr3 | 37053025 | 37053624 | *MLH1* |
| chr5 | 1294968 | 1295567 | *TERT* |
| chr7 | 6048580 | 6049179 | *PMS2* |
| chr12 | 133263828 | 133264427 | *POLE* |
| chr19 | 50887105 | 50887704 | *POLD1* |
| chr19 | 50901590 | 50902189 | *POLD1* |

Table 4: Genes with significant q-values in at least two of three driver-gene prediction algorithms MutSigCV, OncodriveClust and OncodriveFM and a significant combined q-value of the seven driver-prediction tools included in the latest version of intOGen pipeline (see Methods). The second column shows the fraction of samples harboring a point mutation (somatic SNVs or indels) in the respective genes.

| **Gene** | **Mutated samples** | | **MutSigCV** | **OncodriveCLUST** | **OncodriveFM** | **IntOGen combination** |
| --- | --- | --- | --- | --- | --- | --- |
| *BRAF* | 73 | (36.9%) | 0 | 1 × 10^-7^ | 4 × 10^-14^ | 2 × 10^-47^ |
| *NRAS* | 52 | (26.3%) | 0 | 5 × 10^-10^ | 2 × 10^-12^ | 3 × 10^-40^ |
| *NF1* | 47 | (23.7%) | 0 |  | 0 | 8 × 10^-26^ |
| *CDKN2A* | 36 | (18.2%) | 8 × 10^-12^ | 4 × 10^-5^ | 3 × 10^-14^ | 2 × 10^-23^ |
| *TET2* | 30 | (15.2%) | 8 × 10^-12^ |  | 0.007 | 0.0008 |
| *IL7R* | 30 | (15.2%) | 0.097 | 0.04 |  |  |
| *TP53* | 27 | (13.6%) | 0.089 | 0.003 | 0 | 2 × 10^-13^ |
| *ARID2* | 22 | (11.1%) | 8 × 10^-12^ |  | 5 × 10^-7^ | 3 × 10^-7^ |
| *CBL* | 21 | (10.6%) | 8 × 10^-12^ | 0.002 | 0.0006 | 1 × 10^-10^ |
| *NUP98* | 21 | (10.6%) | 1 × 10^-9^ |  | 0.0007 | 0.0009 |
| *DICER1* | 20 | (10.1%) | 0.0001 |  | 0.02 |  |
| *HGF* | 19 | (9.6%) | 5 × 10^-10^ |  | 6 × 10^-5^ |  |
| *SF3B1* | 18 | (9.1%) | 0.097 |  | 4 × 10^-8^ |  |
| *MAP2K1* | 16 | (8.1%) | 2 × 10^-7^ | 0.01 | 3 × 10^-5^ | 8 × 10^-8^ |
| *PPP6C* | 13 | (6.6%) | 0.006 | 1 × 10^-7^ | 1 × 10^-8^ | 3 × 10^-8^ |
| *PTEN* | 10 | (5.1%) | 9 × 10^-5^ |  | 5 × 10^-5^ |  |
| *TCF12* | 10 | (5.1%) | 0.02 |  | 0.03 | 0.001 |
| *CDC27* | 8 | (4.0%) | 0.02 | 0.03 |  |  |
| *RPL5* | 6 | (3.0%) | 0.05 |  | 0.02 | 0.0002 |

Table 5: BRAF mutations not affecting V600/K601 hotspot.

| **Patient ID** | ***BRAF* mutation** | **Molecular subtype** |
| --- | --- | --- |
| 262 | L135P | *NF1* |
| 338 | P328L (splice_region) | *NF1* |
| 324 | P367L | *RAS* |
| 40 | T395I | *RAS* |
| 274 | P420S | *NF1* |
| 286 | G466V | Triple-WT |
| 199 | G469A | Triple-WT |
| 48 | G469R | Triple-WT |
| 112 | N486_P490del | Triple-WT |
| 239 | E501K | *NF1* |
| 178 | N581S | *NF1* |
| 173 | K601_W604del | *BRAF* |
| 297 | 609-3C>T (splice_site) | *NF1* |
| 194 | S616= | *RAS* |
| 301 | Y673D | Triple-WT |
| 227 | Q709* | Triple-WT |
| 193 | P751S | *BRAF* |

Table 6: Limits of top five deleted regions detected by GISTIC.

| **Region** | **Wide Peak Limits** | **Peak Limits** | **Region Limits** |
| --- | --- | --- | --- |
| 9p21.3 | chr9: 21957316-22119516 | chr9: 21967184-22109558 | chr9: 200912-38363920 |
| 6q25.3 | chr6: 156916639-157413840 | chr6: 156926621-157404082 | chr6: 71039367-170624527 |
| 11q23.1 | chr11: 106726859-129025335 | chr11: 110817763-113204048 | chr11: 81264301-135086622 |
| 6q22.1 | chr6: 96766294-129270100 | chr6: 113552503-115153801 | chr6: 71039367-170624527 |
| 10q26.13 | chr10: 121043616-121488137 | chr10: 121479696-121483827 | chr10: 42557507-132615619 |

Table 7: Genes located on the wide-peak of region 11q23.1-3 detected by GISTIC.

| hsa-mir-3167 | NCAM1 | TREH | IFT46 | FAM55A | OR6M1 |
| --- | --- | --- | --- | --- | --- |
| hsa-mir-100 | NFRKB | CEP164 | PRDM10 | FAM55B | OR10G4 |
| hsa-mir-4301 | NNMT | EXPH5 | DSCAML1 | AMICA1 | OR10G7 |
| hsa-mir-34c | NPAT | PHLDB1 | GRAMD1B | CWF19L2 | OR8B3 |
| ACAT1 | NRGN | SIK2 | ARHGAP20 | KDELC2 | OR8A1 |
| ACRV1 | PAFAH1B2 | ARHGEF12 | USP28 | LAYN | C11orf87 |
| APLP2 | POU2AF1 | SIK3 | PKNOX2 | TTC36 | C11orf92 |
| APOA1 | PPP2R1B | VSIG2 | TP53AIP1 | PATE1 | C11orf88 |
| APOA4 | PTS | BACE1 | ABCG4 | C11orf65 | MIR100HG |
| APOC3 | PVRL1 | TRIM29 | ROBO3 | ADAMTS15 | PATE2 |
| ARCN1 | RDX | CADM1 | C11orf1 | MPZL3 | PATE4 |
| ATM | RPS25 | POU2F3 | RNF26 | C11orf45 | FLJ39051 |
| FXYD2 | SC5DL | HINFP | FAM118B | HYLS1 | SNX19 |
| CXCR5 | SCN2B | REXO2 | NLRX1 | TMEM218 | MIRLET7A2 |
| CBL | SCN4B | OR8G2 | C11orf61 | SLC37A2 | MIR100 |
| CD3D | SDHD | OR8B8 | ALG9 | OR8B12 | MIR125B1 |
| CD3E | ST3GAL4 | OR8G1 | CLMP | OR8G5 | MIR34B |
| CD3G | SLN | TIMM8B | PDZD3 | OR10G8 | MIR34C |
| CHEK1 | SORL1 | OR8B2 | C11orf63 | OR10G9 | BLID |
| CRYAB | SRPR | DCPS | CCDC15 | OR10S1 | LINC00167 |
| DDX6 | ST14 | ZBTB44 | TMPRSS5 | OR6T1 | HEPN1 |
| DDX10 | TAGLN | DDX25 | PUS3 | OR4D5 | LOC643923 |
| DLAT | TECTA | NTM | MFRP | TBCEL | CLDN25 |
| DPAGT1 | THY1 | CDON | BCO2 | TMEM136 | LOC649133 |
| DRD2 | UPK2 | SIDT2 | TMPRSS13 | HEPACAM | RPL23AP64 |
| ETS1 | ZBTB16 | TRAPPC4 | KIRREL3 | OAF | LOC100132078 |
| FDX1 | ZNF202 | SPA17 | BUD13 | ANKK1 | PATE3 |
| FLI1 | CUL5 | FXYD6 | TMEM25 | RNF214 | LOC100288346 |
| SLC37A4 | BARX2 | SIAE | RPUSD4 | LOC283143 | BACE1-AS |
| GRIK4 | ZNF259 | C11orf71 | TBRG1 | BCL9L | MIR4301 |
| GUCY1A2 | USP2 | ROBO4 | UBASH3B | FOXR1 | MIR3167 |
| H2AFX | PCSK7 | SLC35F2 | DIXDC1 | CCDC153 | LOC100499227 |
| HMBS | HTR3B | RAB39A | ZC3H12C | OR8D1 | MIR3656 |
| HSPA8 | ZW10 | BTG4 | ESAM | OR8D2 | LOC100507392 |
| HSPB2 | UBE4A | FAM55D | ALKBH8 | OR8B4 | LOC100526771 |
| HTR3A | EI24 | TTC12 | FDXACB1 | KIRREL3-AS3 | HSPB2-C11orf52 |
| IL10RA | FEZ1 | C11orf57 | C11orf52 | CCDC84 | FXYD6-FXYD2 |
| IL18 | ARHGAP32 | ELMOD1 | TIRAP | TMEM225 | MIR4493 |
| STT3A | C2CD2L | FOXRED1 | C1QTNF5 | OR8D4 | MIR4491 |
| KCNJ1 | RBM7 | SCN3B | PANX3 | C11orf53 | MIR4492 |
| KCNJ5 | MPZL2 | VPS11 | APOA5 | LOC341056 | LOC100652768 |
| VWA5A | HYOU1 | TEX12 | TMEM45B | C11orf34 |  |
| MCAM | ATP5L | CRTAM | C11orf93 | BSX |  |
| MLL | ADAMTS8 | TMPRSS4 | PIH1D2 | OR6X1 |  |

Table 8: Potential cancer driver genes located on the wide-peak of region 11q23.1-3 detected by GISTIC.

| **chr** | **symbol** | **cytoband** | **NCG6_oncogene** | **NCG6_tsg** | **cgc_annotation** |
| --- | --- | --- | --- | --- | --- |
| chr11 | ATM | q22.3 | no | yes | TSG |
| chr11 | DDX10 | q22.3 | no | yes | TSG, fusion |
| chr11 | POU2AF1 | q23.1 | yes | no | oncogene, fusion |
| chr11 | SDHD | q23.1 | no | yes | TSG |
| chr11 | ZBTB16 | q23.2 | no | yes | TSG, fusion |
| chr11 | ARHGEF12 | q23.3 | no | yes | TSG, fusion |
| chr11 | BCL9L | q23.3 | no | no | oncogene, TSG |
| chr11 | CBL | q23.3 | no | no | oncogene, TSG, fusion |
| chr11 | DDX6 | q23.3 | yes | no | oncogene, fusion |
| chr11 | FOXR1 | q23.3 | yes | no | oncogene, fusion |
| chr11 | KMT2A | q23.3 | yes | no | oncogene, fusion |
| chr11 | PAFAH1B2 | q23.3 | no | no | fusion |
| chr11 | FLI1 | q24.3 | yes | no | oncogene, fusion |
| chr11 | KCNJ5 | q24.3 | yes | no | oncogene |

Table 9: Somatic mutations in CBL: number of patients affected SNVs or CNVs in CBL.

| **Subtype** | **Samples** | **SNV** | **CNV** | **SNV or CNV** | **SNV and CNV** |
| --- | --- | --- | --- | --- | --- |
| **BRAF** | 58 | 2 (3%) | 12 (21%) | 14 (24%) | 0 (0%) |
| **NF1** | 35 | 12 (34%) | 14 (40%) | 21 (60%) | 5 (14%) |
| **RAS** | 54 | 2 (4%) | 25 (48%) | 26 (48%) | 1 (2%) |
| **TripleWT** | 46 | 6 (13%) | 15 (33%) | 20 (43%) | 1 (2%) |
| **all** | 193 | 22 (11%) | 66 (34%) | 81 (42%) | 7 (4%) |

Table 10: Somatic SNVs in *CBL* in TCGA and MSK cohorts. Patients affected by SNV and CNV in *CBL* were enriched in NF1 subtype (highlighted in the table).

| **Cohort** | **Subtype** | **Samples** | **SNVs in CBL** | **SNV in CBL %** |
| --- | --- | --- | --- | --- |
| Skin Cutaneous Melanoma TCGA PanCancer Atlas | All | 363 | 17 | 4.68% |
|  | BRAF exclusive | 151 | 4 | 2.65% |
|  | NF1 exclusive | 27 | 4 | 14.81% |
|  | *RAS exclusive | 92 | 6 | 6.52% |
|  | TripleWT | 41 | 1 | 2.44% |
|  |  |  |  |  |
| Melanoma (MSKCC, Clin Cancer Res 2021) | All | 556 | 62 | 11.15% |
|  | BRAF exclusive | 190 | 6 | 3.16% |
|  | NF1 exclusive | 86 | 26 | 30.23% |
|  | *RAS exclusive | 137 | 9 | 6.57% |
|  | TripleWT | 65 | 10 | 15.38% |

Table 11: Cancer driver gene prediction using 7 methods included in the intOGen pipeline

| **Mutation count bias** |  |
| --- | --- |
| CBaSE | \| gene \| p_pos \| q_pos \| mis_obs \| non_obs \| syn_obs \| \| --- \| --- \| --- \| --- \| --- \| --- \| \| CBL \| 2.491244e-04 \| 1.603387e-01 \| 17 \| 2 \| 1 \| |
| MutPanning | \| Name \| TargetSize \| TargetSizeSyn \| Count \| CountSyn \| Significance \| FDR \| \| --- \| --- \| --- \| --- \| --- \| --- \| --- \| \| CBL \| 6112.051027317315 \| 2153.252141234597 \| 20 \| 2 \| 0.0012670730910869787 \| 0.12580225690077862 \| |
| dNdScv | \| gene_name \| n_syn \| n_mis \| n_non \| n_spl \| n_ind \| wmis_cv \| wnon_cv \| wspl_cv \| wind_cv \| pmis_cv \| ptrunc_cv \| pallsubs_cv \| pind_cv \| qmis_cv \| qtrunc_cv \| qallsubs_cv \| pglobal_cv \| qglobal_cv \| \| --- \| --- \| --- \| --- \| --- \| --- \| --- \| --- \| --- \| --- \| --- \| --- \| --- \| --- \| --- \| --- \| --- \| --- \| --- \| \| CBL \| 2 \| 17 \| 2 \| 1 \| 0 \| 27.0077001468087 \| 47.6049820189567 \| 47.6049820189567 \| 0 \| 0 \| 3.1865097866457e-05 \| 0 \| 1 \| 0 \| 0.00961529876790991 \| 0 \| 0 \| 0 \| |
|  |  |
| **Mutation clustering in protein** |  |
| OncodriveCLUSTL | \| RANK \| SYMBOL \| ENSID \| CGC \| CHROMOSOME \| STRAND \| COORDINATES \| MAX_COORD \| WIDTH \| N_MUT \| N_SAMPLES \| FRA_UNIQ_SAMPLES \| SCORE \| P \| \| --- \| --- \| --- \| --- \| --- \| --- \| --- \| --- \| --- \| --- \| --- \| --- \| --- \| --- \| \| 60 \| CBL \| ENSG00000110395 \| Non Available \| 11 \| + \| 119278531,119278535 \| 119278533 \| 5 \| 7 \| 5 \| 0.7142857142857143 \| 144.2809 \| 0.0335065525336235 \| \| 60 \| CBL \| ENSG00000110395 \| Non Available \| 11 \| + \| 119278171,119278181 \| 119278181 \| 11 \| 3 \| 3 \| 1.0 \| 29.0179 \| 0.5473256623962748 \| \| 60 \| CBL \| ENSG00000110395 \| Non Available \| 11 \| + \| 119277821,119277837 \| 119277836 \| 17 \| 3 \| 2 \| 0.6666666666666666 \| 24.4662 \| 0.5865760199136167 \| \| 60 \| CBL \| ENSG00000110395 \| Non Available \| 11 \| + \| 119271829,119271829 \| 119271829 \| 1 \| 2 \| 2 \| 1.0 \| 19.0476 \| 0.6331214002644805 \| |
| HotMAPS | \| GENE \| Cancer_Type \| TOTAL_HSP \| TOTAL_RES_HSP \| MEDIAN_RES_HSP \| Min p-value \| q-value \| \| --- \| --- \| --- \| --- \| --- \| --- \| --- \| \| CBL \| Melanoma1 \| 1 \| 3 \| 3.0 \| 6.943383649720183e-06 \| 0.001870138580747896 \|  \| CRAVAT Res \| HUGO Symbol \| Min p-value \| Ref AA \| Sequence Ontology Transcript \| Tumor Type \| chromosome \| genomic position \| genomic position hg19 \| q-value \| \| --- \| --- \| --- \| --- \| --- \| --- \| --- \| --- \| --- \| --- \| \| 180 \| CBL \| 0.05745972800722636 \| R \| ENST00000264033 \| Melanoma1 \| chr11 \| 119271829,119271830,119271831 \| 119142539,119142540,119142541 \| 0.7096902674700801 \| \| 217 \| CBL \| 0.0002985654969379678 \| S \| ENST00000264033 \| Melanoma1 \| chr11 \| 119273926,119273927,119273928 \| 119144636,119144637,119144638 \| 0.02447490661148991 \| \| 363 \| CBL \| 0.02409354126452903 \| V \| ENST00000264033 \| Melanoma1 \| chr11 \| 119277836,119277837,119277838 \| 119148546,119148547,119148548 \| 0.4674717266650337 \| \| 367 \| CBL \| 0.003171474770467116 \| Q \| ENST00000264033 \| Melanoma1 \| chr11 \| 119278169,119278170,119278171 \| 119148879,119148880,119148881 \| 0.1299908221545209 \| \| 371 \| CBL \| 0.1242218783180774 \| Y \| ENST00000264033 \| Melanoma1 \| chr11 \| 119278181,119278182,119278183 \| 119148891,119148892,119148893 \| 0.9098992194216108 \| \| 417 \| CBL \| 6.943383649720183e-06 \| P \| ENST00000264033 \| Melanoma1 \| chr11 \| 119278531,119278532,119278533 \| 119149241,119149242,119149243 \| 0.001870138580747896 \| \| 418 \| CBL \| 6.943383649720183e-06 \| F \| ENST00000264033 \| Melanoma1 \| chr11 \| 119278534,119278535,119278536 \| 119149244,119149245,119149246 \| 0.001870138580747896 \| |
| smRegions | \| REGION \| HUGO_SYMBOL \| TOTAL_MUTS_GENE \| OBSERVED_REGION \| MEAN_SIMULATED \| U \| P_VALUE \| Q_VALUE \| \| --- \| --- \| --- \| --- \| --- \| --- \| --- \| --- \| \| ENST00000264033:PF02761:179:262 \| CBL \| 20 \| 3 \| 1.82 \| 0.7169574059104686 \| 0.3971437522828911 \| 0.8408953850185598 \| \| ENST00000264033:PF14447:381:429 \| CBL \| 20 \| 7 \| 0.962 \| 17.86643544964742 \| 2.369637986718025e-05 \| 0.007042208165528983 \| \| ENST00000264033:PF00627:858:892 \| CBL \| 20 \| 1 \| 0.954 \| 0.002294404061975855 \| 0.9617959887913655 \| 0.9857591957601198 \| |
|  |  |
| **Functional impact bias** |  |
| OncodriveFML | \| GENE_ID \| MUTS \| MUTS_RECURRENCE \| SAMPLES \| P_VALUE \| Q_VALUE \| P_VALUE_NEG \| Q_VALUE_NEG \| SNP \| MNP \| INDELS \| SYMBOL \| \| --- \| --- \| --- \| --- \| --- \| --- \| --- \| --- \| --- \| --- \| --- \| --- \| \| ENSG00000110395 \| 21 \| 16 \| 18 \| 0.00211 \| 0.137783 \| 0.99789 \| 1.0 \| 21 \| 0 \| 0 \| CBL \| |

## Supplementary Figures

Supplementary Figure 1: (A) Driver variants in CBL identified in cBioPortal cluster in a zinc finger domain. (B) CBL harbours mutations in ˜9% of patients in 9 studies included in cBioPortal. (C) In TCGA PanCancerAtlas, the frequency of CBL mutations is ~7%.


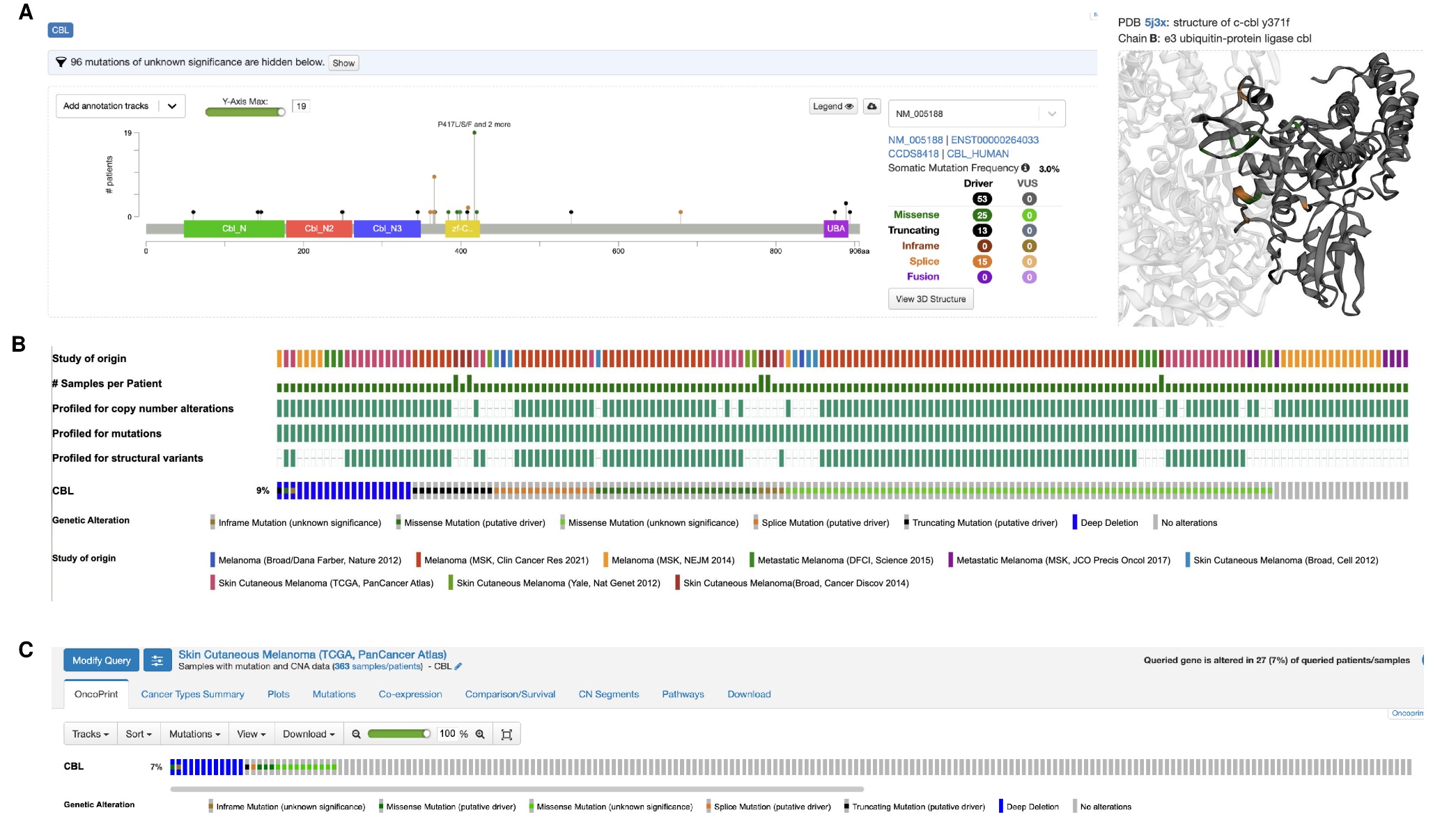


Supplementary Figure 2: Improved OS of BRAF-subtype compared to Triple-WT (not significant, p=0.06).


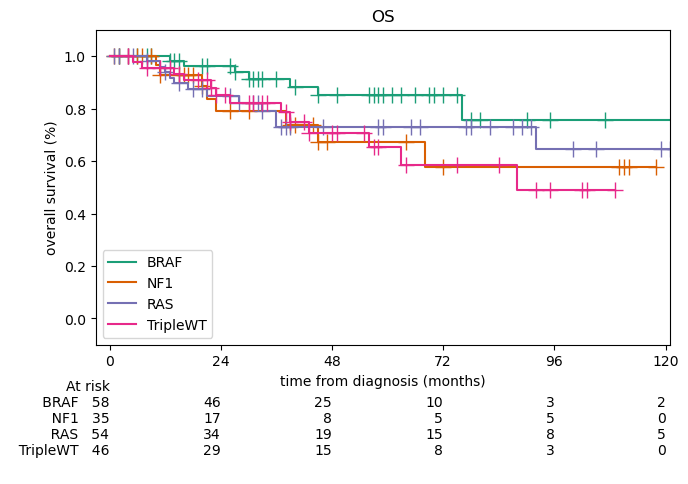


Supplementary Figure 3: The effect of the CBL deletion on RFS is not significant across the whole cohort and in the *BRAF*, *NF1* and Triple-WT molecular subtypes.


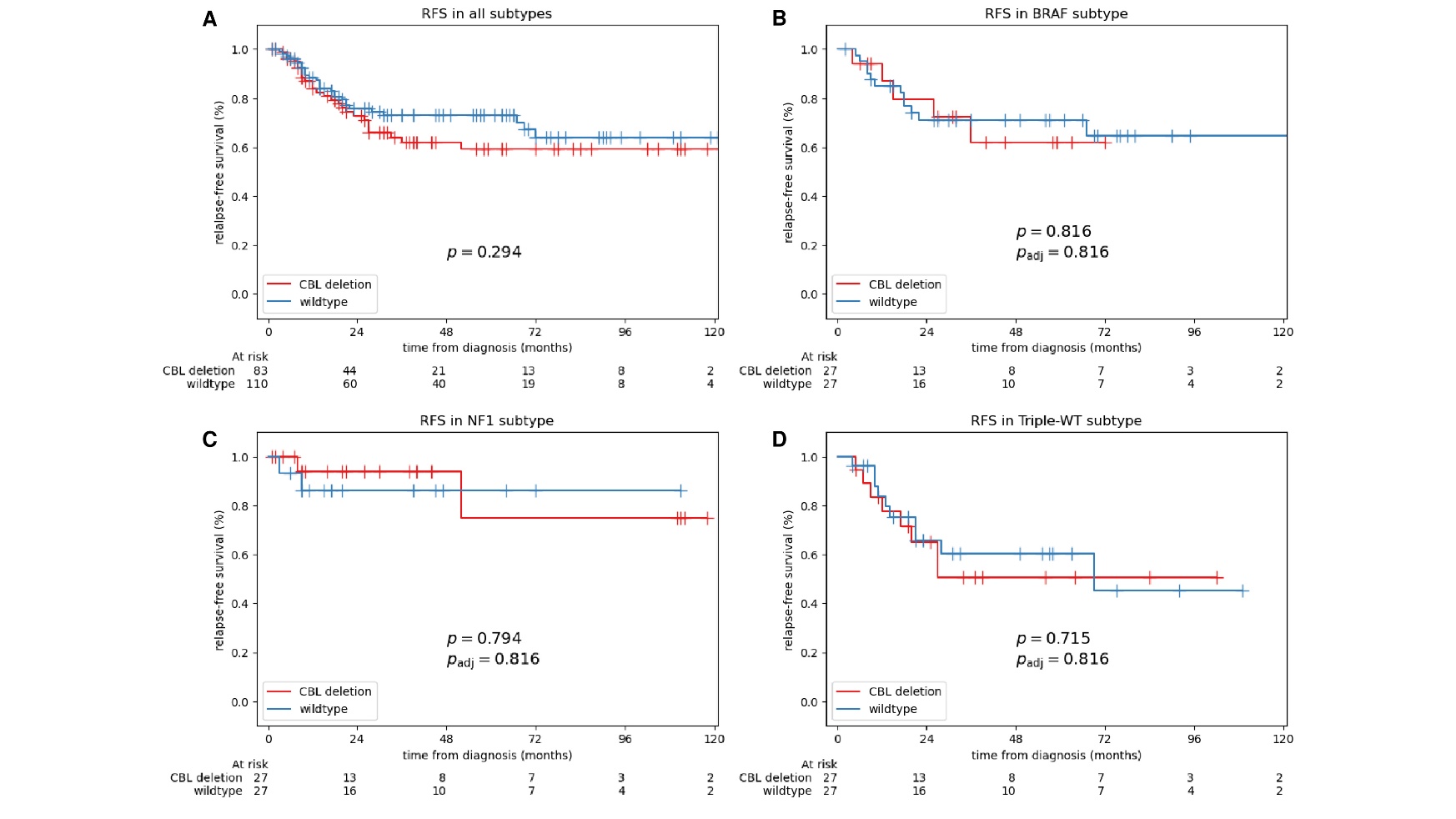

Supplement: Supplementary file 1 — Supplementary Material [file 41416_2026_3394_MOESM1_ESM.docx]
